# An isothermal calorimetry assay for determining steady state kinetic and Ensitrelvir inhibition parameters for SARS-CoV-2 3CL-protease

**DOI:** 10.1038/s41598-024-81990-y

**Published:** 2024-12-31

**Authors:** Luca Mazzei, Sofia Ranieri, Davide Silvestri, Rebecca Greene-Cramer, Christopher Cioffi, Gaetano T. Montelione, Stefano Ciurli

**Affiliations:** 1 Laboratory of Bioinorganic Chemistry, Department of Pharmacy and Biotechnology, University of Bologna, 40127 Bologna, Italy; 2 Center for Biotechnology and Interdisciplinary Sciences, Rensselaer Polytechnic Institute, Troy, NY 12180 USA; 3 Department of Chemistry and Chemical Biology, Rensselaer Polytechnic Institute, Troy, NY 12180 USA

**Keywords:** Enzyme mechanisms, Proteases

## Abstract

This manuscript details the application of Isothermal Titration Calorimetry (ITC) to characterize the kinetics of 3CL^pro^, the main protease from the Severe Acute Respiratory Syndrome CoronaVirus-2 (SARS-CoV-2), and its inhibition by Ensitrelvir, a known non-covalent inhibitor. 3CL^pro^ is essential for producing the proteins necessary for viral infection, which led to the COVID-19 pandemic. The ITC-based assay provided rapid and reliable measurements of 3CL^pro^ activity, allowing for the direct derivation of the kinetic enzymatic constants *K*_*M*_ and *k*_*cat*_ by monitoring the thermal power required to maintain a constant temperature as the substrate is consumed. The manuscript highlights several advantages of the proposed ITC-based assay over traditional methods used to study 3CL^pro^, such as Förster Resonance Energy Transfer (FRET) and Liquid Chromatography-Mass Spectrometry (LC–MS) and overcomes the need for non-biological substrates or discontinuous post-reaction steps. The ease of application of the ITC method allowed for the determination of the temperature dependence of the catalytic constants, enabling the estimation of the reaction activation energy. Additionally, the assay was used to determine the inhibition mode and kinetic parameters for 3CL^pro^ inhibition by Ensitrelvir. This molecule was revealed to act as a slow- and tight-binding inhibitor that forms an initial E•I complex (*K*_*I*_ = 9.9 ± 0.7 nM) quickly transitioning to a tighter E•I* assembly (*K*_I_* = 1.1 ± 0.2 nM). This versatile calorimetric method is proposed for general use in the discovery and development of drugs targeting 3CL^pro^.

**Supplementary Information:**

The online version contains supplementary material available at 10.1038/s41598-024-81990-y.

## Introduction

Severe Acute Respiratory Syndrome CoronaVirus-2 (SARS-CoV-2), the causative agent of coronavirus disease 2019 (COVID-19), has been responsible for over seven million deaths worldwide, posing a significant threat to the global economy and healthcare system^1^. During SARS-CoV-2 infection, the viral proteases 3CL^pro^ and PL^pro^ are crucial for catalyzing the hydrolysis of two polyproteins, pp1a and pp1ab, which are translated by host ribosomes upon recognition of the viral positive single-stranded RNA^2,3^. The enzymatic cleavage of pp1a and pp1ab releases a set of non-structural viral proteins essential for SARS-CoV-2 replication^4,5^.

**Fig. 1:**
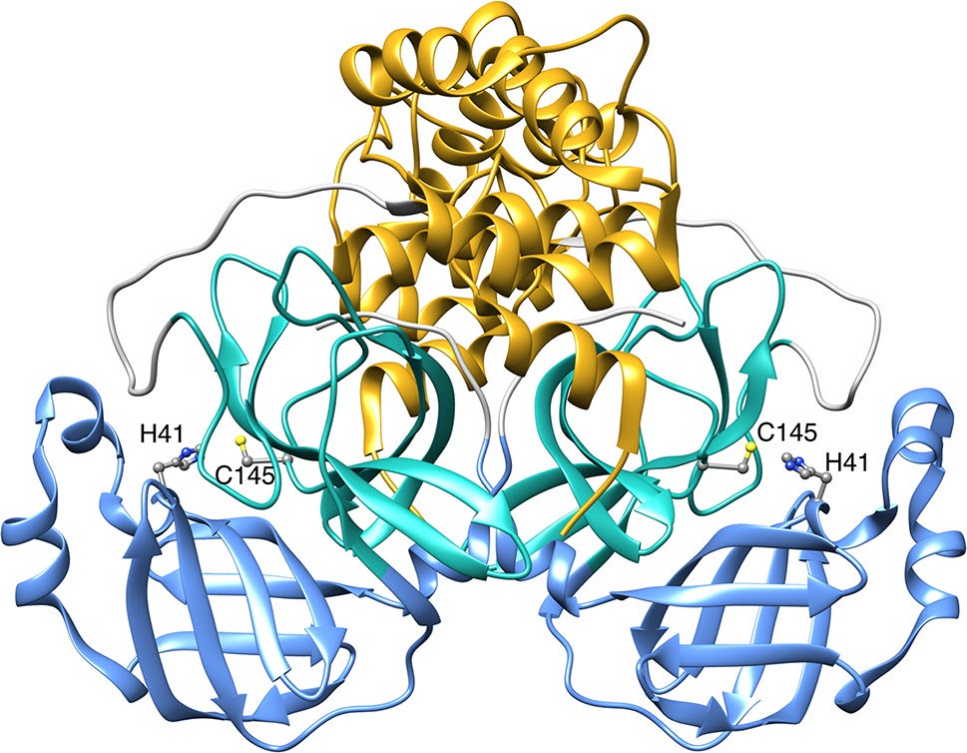
Ribbon representation of SARS-CoV-2 3CL^pro^ (PDB code 8CDC^18^). Domains I, II, and III are colored in cornflower blue, light sea green and goldenrod, respectively. The side chains of the H41—C145 catalytic dyad are shown as ball-and-stick and colored according to the CPK code. Figure made using Chimera^19^.

SARS-CoV-2 3CL^pro^ is a homodimeric cysteine PA-clan protease^6^, composed of two 33.8-kDa protomers, each consisting of three structural domains (Fig. 1): domain I (residues 8 – 101), domain II (residues 102 – 184), and domain III (residues 201 – 306). The active site cleft, containing the His41—Cys145 (H41-C145) catalytic dyad, is located between domains I and II and comprises multiple subsites (S4, S3, S2, S1, and S1’). During catalysis, these subsites are occupied by specific sequences of substrate amino acid residues (P4, P3, P2, P1, and P1’, respectively). The substrate recognition motif, highly conserved among several coronavirus 3CL^pro^, prefers the Leu-Gln-Ser (LQS) sequence in the P2-P1-P1’ position^7^.

3CL^pro^ exhibits minimal catalytic activity in its monomeric state, with dimerization deemed essential for full protease activity^8,9^. Crystallographic evidence indicates that the dimerization process involves the first seven N-terminal residues of each protomer (N-fingers), which contribute to dimer stabilization and active site architecture, particularly the S1 subsite, through interactions with domain II of the adjacent protomer and domains II and III of the parent protomer^10^. Conversely, solution studies using native mass spectroscopy suggest that N-terminal processing is not critical for dimerization that, instead, appears to be triggered by an induced fit resulting from covalent linkage between the active site cysteine thiol S atom and the carbonyl C of the substrate during catalysis^8^.

Given its crucial role in the viral life cycle and the absence of closely related homologs in the human genome, drug discovery targeting 3CL^pro^ has been a pivotal research area during the SARS-CoV-2 pandemic. Several 3CL^pro^ inhibitors have been identified^11–14^, which can be categorized into two major families: peptidomimetics and non-peptide small molecules. Peptidomimetics are designed based on natural substrate scaffolds and typically share with them the same binding subsites. These inhibitors usually exploit an electrophilic warhead group (*i.e.*, aldehydes, ketones, Michael acceptors, and nitriles) near the P1 moiety to covalently bind the nucleophilic thiol group of C145, thereby inactivating 3CL^pro^. Non-peptide small molecules inhibitors of 3CL^pro^ encompass a diverse range of compounds, including flavonoids, terpenoids, quinoline analogs, pyridinyl esters, Ebselen analogs, benzotriazole-based compounds, pyrimidine analogs, acrylamide and related compounds, isatin analogs, triazine compounds, and metal-containing analogs. Additionally, certain macrocyclic inhibitors of other PA-clan proteases have also been found to inhibit SARS CoV-2 3CL^pro^^15–17^.

Despite the critical role of 3CL^pro^ in the spread of COVID-19, detailed kinetic data on its activity and inhibition remain limited and often highly variable. This inconsistency arises from differences in methodologies, the form of 3CL^pro^ used in assays, and the substrates analyzed^20,21^. The two most common methods for characterizing 3CL^pro^ kinetics are Förster Resonance Energy Transfer (FRET)^20–26^ and Liquid Chromatography-Mass Spectrometry (LC–MS)^27,28^. The kinetic parameters* K*_*M*_ and *k*_*cat*_ obtained by these techniques span several orders of magnitude. For instance, *K*_*M*_ values reported using FRET range from 17 to 60 µM for SARS-CoV 3CL^pro^^21,28,29^ and 28 to 230 µM for SARS-CoV-2 3CL^pro^^21,30,31^. In contrast, results based on LC–MS show *K*_*M*_ values in the range of 0.2 to 2.6 mM for SARS-CoV 3CL^pro^^21,32^ and 0.9 mM for 3CL^pro^ from SARS-CoV-2^21^. Similarly, *k*_*cat*_ values obtained by FRET range from 0.2 to 2 s^-1^ for SARS-CoV 3CL^pro^^21,28,29^ and 0.05 to 0.23 s^-1^ for SARS-CoV-2 3CL^pro^^21,30,31^, while LC–MS yields *k*_*cat*_ values of 0.54 to 6.4 s^-1^ for SARS-CoV 3CL^pro^^21,32^ and 2.2 s^-1^ for SARS-CoV-2 3CL^pro^^21^. Thus, the *K*_*M*_ values estimated by LC–MS (in the mM range) are generally much higher than those reported for FRET-based methods (in the sub-millimolar range), while the *k*_*cat*_ values span at least two orders of magnitude independently of the technique used.

These discrepancies reveal less-than-optimal reliability for both types of assays. Additionally, each method presents significant disadvantages: LC–MS is a discontinuous method requiring several time-consuming sample manipulation steps, potentially leading to non-negligible experimental errors. Furthermore, FRET-based methods are susceptible to the inner filter effect, where fluorescent light is absorbed by quenching groups on neighboring substrates or cleaved reaction products, resulting in only a fraction of the fluorescence reaching the detector system of the fluorimeter^33,34^. FRET can also be affected by interactions between the substrate fluorophore and the enzyme, as well as by the absorbance or fluorescence of the inhibitor itself. These drawbacks highlight the need for alternative approaches that can rapidly and effectively evaluate the catalytic and inhibitory efficiency of SARS-CoV-2 3CL^pro^ and related PA-clan proteases. Such improvements are crucial for optimizing drug screening and optimization processes, as well as for gaining deeper insights into the fundamental mechanisms of enzyme inhibition.

Isothermal Titration Calorimetry (ITC) is a technique used to characterize enzyme kinetics by monitoring the heat generated upon rapid mixing of small-volume injections of a substrate (or enzyme) solution into a sample cell containing an enzyme (or substrate) solution, either in the absence or presence of varying concentrations of an inhibitor^35–41^. This approach is highly versatile, as most chemical reactions involve heat production or consumption. Unlike other techniques that infer catalysis rates indirectly from substrate or product concentrations, ITC provides real-time detection of heat flow, offering a direct measurement of enzyme activity and its modulation by inhibitors. ITC does not require the development of customized assays using fluorophores or chromophores as substrates or products as needed for FRET, nor does it require post-reaction separation of products and substrates, as with LC–MS. Additionally, compared to reported measurements where enzyme, substrate, and inhibitor solutions are combined with different incubation times before the measurement, ITC measures heat flow rapidly, minimizing dead time. Despite its considerable potential, no study to date has utilized ITC to characterize the kinetics of catalysis and inhibition of 3CL^pro^.

**Fig. 2:**
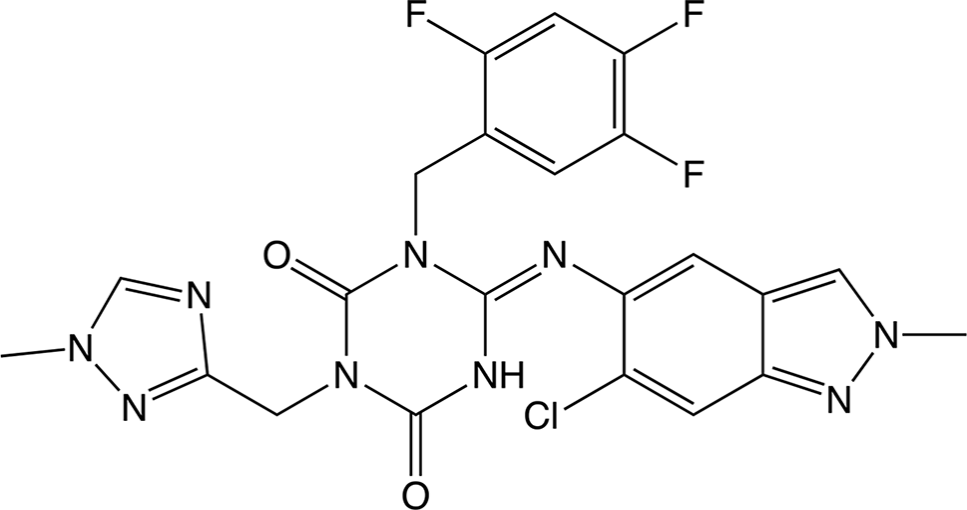
Chemical structure of Ensitrelvir.

Here we provide a comprehensive characterization of the activity of 3CL^pro^ from SARS-CoV-2 using ITC. This method delivers kinetic parameters in less than an hour, with high reliability. Additionally, we describe the application of ITC to study the inhibition of 3CL^pro^ by Ensitrelvir, a well-established 3CL^pro^ inhibitor. Ensitrelvir is the first oral non-covalent and non-peptide inhibitor from the triazine compound family (Fig. 2), developed by Shionogi^42^. It was approved for emergency use in Japan in November 2022 and is marketed under the brand name Xocova.

## Results

### General description of the methodology

**Fig. 3:**
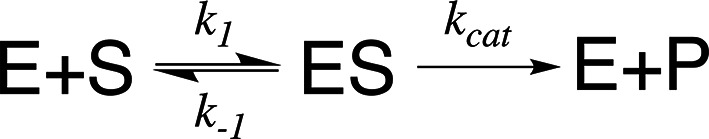
Classical enzymatic reaction.

The most used approach to mathematically describe a classical enzyme-catalyzed reaction (Fig. 3) is the Michaelis–Menten model (Eq. 1). This model describes the reaction rate, expressed as the time-dependent decrease of substrate concentration [S], as a function of [S], the enzyme concentration [E], and the parameters *K*_*M*_ and *k*_*cat*_.1

In Eq. 1, *K*_*M*_ (commonly known as Michaelis constant) is the pseudo-equilibrium constant [(*k*_*-1*_ + *k*_*cat*_*)/k*_*1*_] under steady-state conditions, and it represents the substrate concentration required to achieve half-maximal reaction rate. *k*_*cat*_ is the catalytic rate constant (also known as turnover number) describing the limiting number of substrate molecules converted per second by the enzyme. The term *k*_*cat*_ •[E], also defined as *V*_*max*_, is the maximum reaction rate theoretically achieved in the presence of an infinite amount of substrate. Complete characterization of any enzyme-catalyzed reaction involves determining *K*_*M*_ and *k*_*cat*_. To achieve this objective, the reaction can be studied using ITC^35–41^, as briefly described here.

**Fig. 4:**
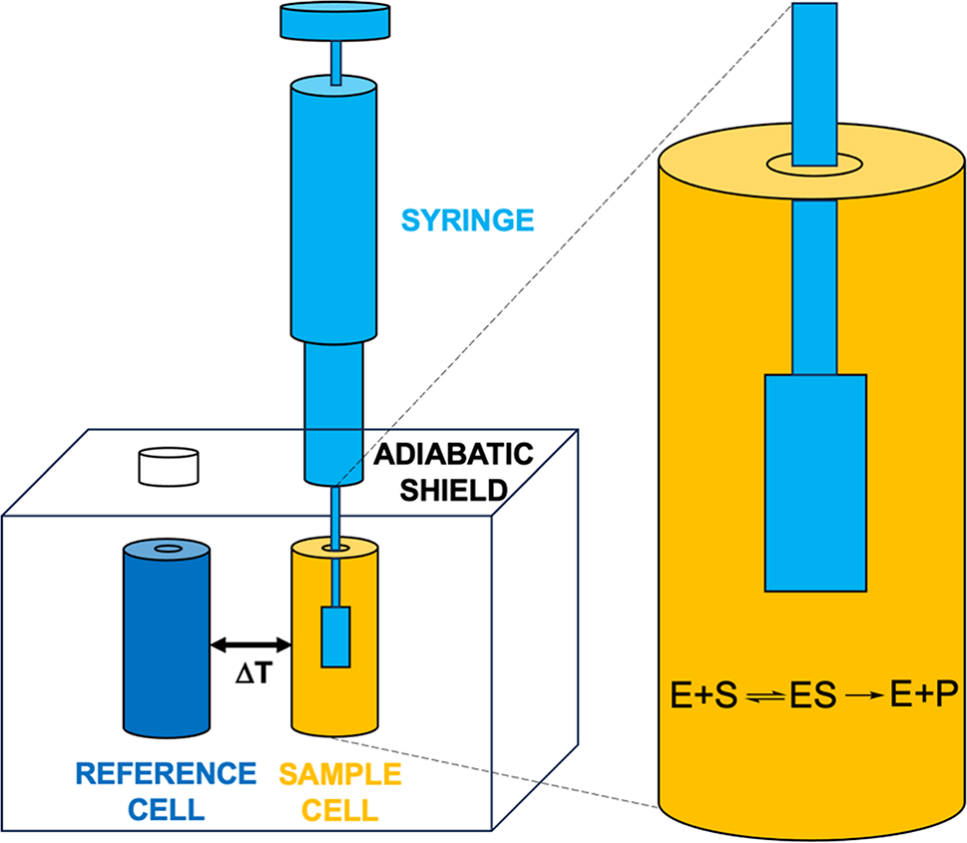
Schematic representation of the isothermal titration calorimeter: reference and sample cells are indicated in dark blue and orange, respectively, and the titration syringe is colored in light blue.

The isothermal calorimeter (Fig. 4) consists of an adiabatic shield encompassing two cells: a reference cell (usually filled with deionized water) and a sample cell. A computer-controlled syringe is mounted on the sample cell, where it dispenses its content using a rotating, paddle-shaped needle that ensures complete mixing of the solutions after each injection. During the ITC experiment, a thermoelectric device continuously measures the temperature difference between the sample and reference cells and, using a cell feedback network, it maintains this difference (ΔT) at zero by adding or removing heat from the sample cell.

The amount of heat (*Q*) added or removed by the system over time (*t*) is defined as the thermal power (*TP*), often also referred to as heat flow (Eq. 2):2

In an enzyme-catalyzed reaction, the heat associated with the conversion of *n* moles of substrate to product at constant pressure is described by Eq. 3:3

Here, *ΔH*_*app*_ represents the total apparent molar enthalpy for the reaction, [S] is the molar concentration of converted substrate, and *V*_*cell*_ is the volume of the sample cell where the reaction occurs. The reaction rate, defined as the change in substrate concentration over time, can be related to the thermal power by Eq. 4:4

Therefore, to calculate the reaction rates as a function of the substrate concentration, which then can be fitted using the Michaelis–Menten equation (Eq. 1) to derive the kinetic parameters *K*_*M*_ and *k*_*cat*_, two key components are required: (i) knowledge of the total apparent molar enthalpy *ΔH*_*app*_ and (ii) measurements of *dQ/dt* at various substrate concentrations.

*ΔH*_*app*_ is typically determined using the *direct single-injection* setup. In this method, a small amount of substrate, at a final concentration smaller than the expected value of *K*_*M*_, is injected into a solution containing the enzyme in the nM–μM range. The thermal power generated by the reaction is monitored over time until the substrate is completely consumed and the signal returns to the pre-injection baseline. The enzyme and substrate concentrations are chosen so that complete conversion occurs within minutes. *ΔH*_*app*_ is thus calculated by integrating the area under the curve, according to Eq. 5, where [S]_total_ is the total concentration of substrate present in the sample cell at the start of the experiment:5

The determination of *dQ/dt* values at various substrate concentrations typically employs the *multiple-injection* method. This involves sequential small injections of concentrated substrate solution into a diluted (in the pM–nM range) enzyme solution. Each injection increases the substrate concentration, causing a shift in the baseline that reflects a change in thermal power within the sample cell. These injections are timed to allow the thermal power to stabilize at the new baseline level but must be short enough to prevent significant substrate conversion (less than 5%), ensuring measurements are conducted under steady-state conditions. The *dQ/dt* value at each substrate concentration can thus be determined by measuring the difference between the original baseline and the new baseline after each injection. Using Eq. 4, the resulting reaction rates as a function of substrate concentration can then be computed and fitted to Eq. 1 to derive the kinetic parameters *K*_*M*_ and *k*_*cat*_.

A drawback of the *multiple-injection* method is the typically low total thermal power generated, especially if the reaction has a small *ΔH*_*app*_. This can make the assay sensitive to baseline drifts and instrumental noise, potentially affecting the reliability of *K*_*M*_ and *k*_*cat*_ estimates. Additionally, achieving high substrate concentrations in the syringe solution may be challenging for sparingly soluble compounds, while the large heat of substrate dilution could significantly interfere with heat of reaction measurements. In our investigation of 3CL^pro^ enzymatic activity, we encountered these typical challenges of the *multiple-injection* method and thus explored the *inverse single-injection* method as an alternative approach, described below.

### Derivation of the kinetic parameters of 3CL^pro^ enzymatic catalysis

**Fig. 5:**
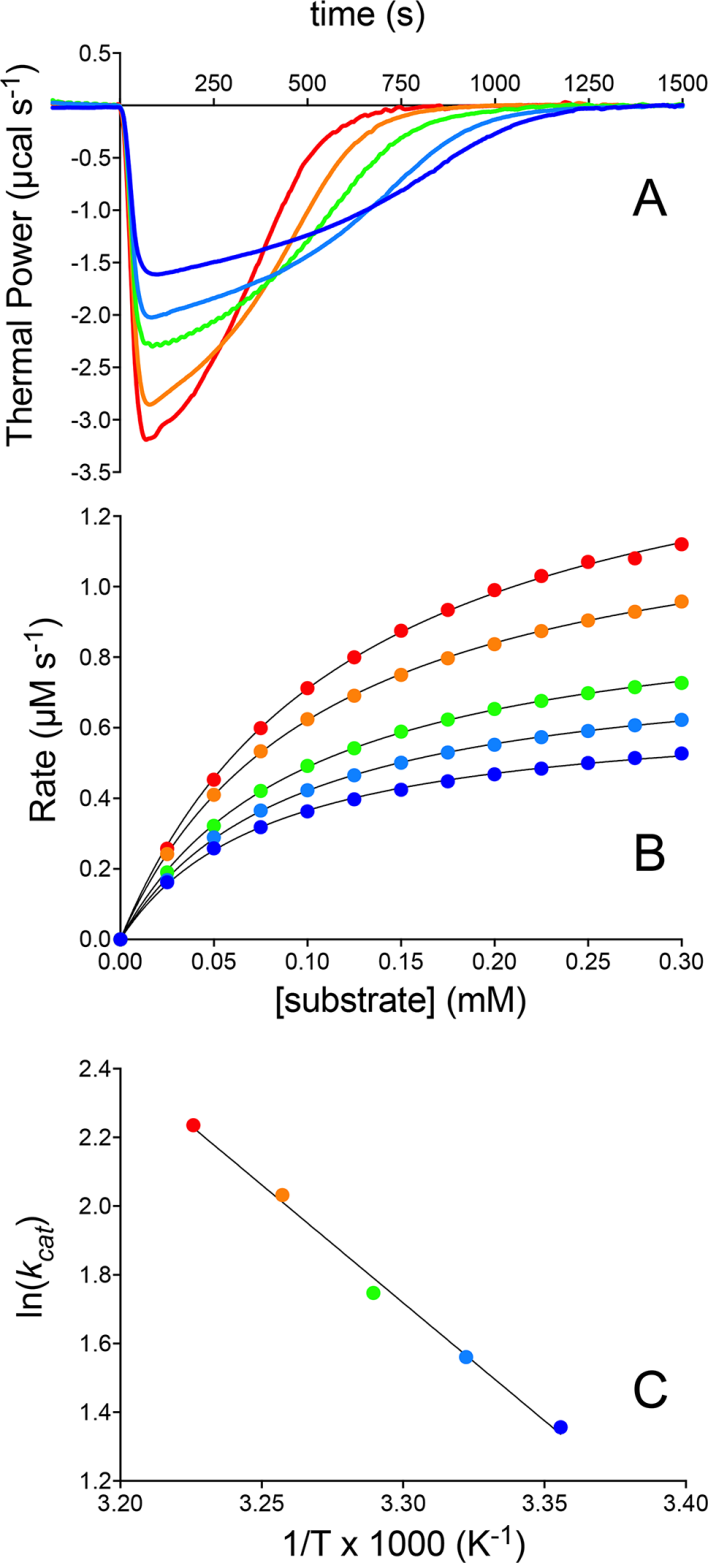
3CL^pro^ kinetics at pH 7.5 characterized by ITC. (**A**) Thermal power recorded over time following injection of 150 nM 3CL^pro^ into a sample cell containing 0.35 mM substrate at 298 K (dark blue line), 301 K (cyan line), 304 K (green line), 307 K (orange line), and 310 K (red line). (**B**) Corresponding reaction rates calculated using Eq. 4 and fits to the Michaelis–Menten equation shown as colored lines. The reaction rates represent a subset of the experimentally measured data points (a few hundred in total), selected every 0.025 mM for clarity. (**C**) Arrhenius plot depicting the logarithm of the *k*_*cat*_ values against the inverse temperature, 1/T, used to determine the activation energy. In panels (**B**,**C**), the color-code corresponds to that in (**A**).

Using the *inverse single-injection* method, a small volume of 3CL^pro^ solution (in the tens of µM range) is injected into the sample cell containing a substrate solution (*ca.* 1.5 mL) at a concentration significantly higher than the expected *K*_*M*_. This approach ensures substantial enzyme saturation while avoiding solubility issues. The rapid hydrolysis of the concentrated substrate upon enzyme injection leads to a marked decrease in the thermal power *dQ/dt* (Fig. 5A), indicative of an exothermic reaction. This decrease reaches its maximal effect and then gradually returns to the pre-injection baseline as the substrate is depleted (Fig. 5A). The heat of mixing, measured separately by injecting enzyme solution into buffer alone, is negligible. Integration of these data using Eq. 5 yields *ΔH*_*app*_. The change in substrate concentration over any time interval can be calculated using Eq. 6:6

In this equation, *t*_*1*_ and *t*_*2*_ denote two consecutive time points (typically separated by 2 s). Reaction rates as a function of substrate concentration are then derived using Eq. 4 and fitted to the Michaelis–Menten equation (Eq. 1) to determine *K*_*M*_ and *k*_*cat*_.

Using this methodology, the kinetics of 3CL^pro^ were studied at five different temperatures ranging from 298 to 310 K (Fig. 5). At 298 K, the obtained values were *ΔH*_*app*_ = -2.1 kcal mol^-1^, *K*_*M*_ = 81 ± 2 µM and *k*_*cat*_ = 3.9 ± 0.1 s^-1^. These values are fully consistent with those previously reported for SARS-CoV-2 3CL^pro^ using a similar calorimetric method, but a different data treatment^43^.

The measured values of *ΔH*_*app*_ are invariant with respect to temperature (Table 1) and consistent with values reported for peptide bond hydrolysis under similar buffer and temperature conditions^44^. *K*_*M*_ increases slightly with temperature, from 81 to 124 µM, indicating a modest reduction in enzyme affinity for the substrate at higher temperatures, while *k*_*cat*_ significantly increases from 3.9 to 9.3 s^-1^ in the explored temperature range. The temperature dependence of *k*_*cat*_ allowed for the determination of the activation energy (*E*_*a*_ = 13.1 ± 1.4 kcal mol^-1^) for the 3CL^pro^ hydrolytic reaction, derived from the Arrhenius equation (Eq. 7) and the corresponding Eyring plot (Fig. 5C):7

**Table 1:** Kinetic parameters of wild type 3CL^pro^ measured at different temperatures.

	*ΔH*_*app*_ (kcal mol^-1^)	*K*_*M*_ (µM)	*k*_*cat*_ (s^-1^)	*k*_*cat*_*/K*_*M*_ (M^-1^ s^-1^)
298 K	-2.1 ± 0.1	81 ± 2	3.9 ± 0.1	(48 ± 2) × 10^3^
301 K	-2.2 ± 0.1	92 ± 1	4.8 ± 0.1	(52 ± 2) × 10^3^
304 K	-2.2 ± 0.1	100 ± 1	5.7 ± 0.1	(57 ± 2) × 10^3^
307 K	-2.0 ± 0.1	109 ± 1	7.6 ± 0.1	(70 ± 2) × 10^3^
310 K	-2.0 ± 0.1	124 ± 3	9.3 ± 0.1	(75 ± 3) × 10^3^

### Derivation of the inhibition mode of 3CL^pro^ by Ensitrelvir using ITC

The *inverse single-injection* method is also adaptable for studying enzyme inhibition kinetics by introducing inhibitors into the substrate solution at saturating concentration in the sample cell prior to enzyme addition. We applied this approach to investigate the inhibition kinetics of 3CL^pro^ by the non-covalent inhibitor Ensitrelvir (Fig. 7). Raw data (Fig. 7A) were collected from experiments with varying concentrations of Ensitrelvir (in the 12.5 – 250 nM range), encompassing the reported IC_50_ value (*ca.* 50 nM^45^). The results showed that the thermal power initially decreased upon enzyme injection to a level dependent on the inhibitor concentration, eventually returning slowly towards the pre-injection baseline without fully reaching it unless the inhibitor concentration significantly exceeded the enzyme concentration.

**Fig. 6:**
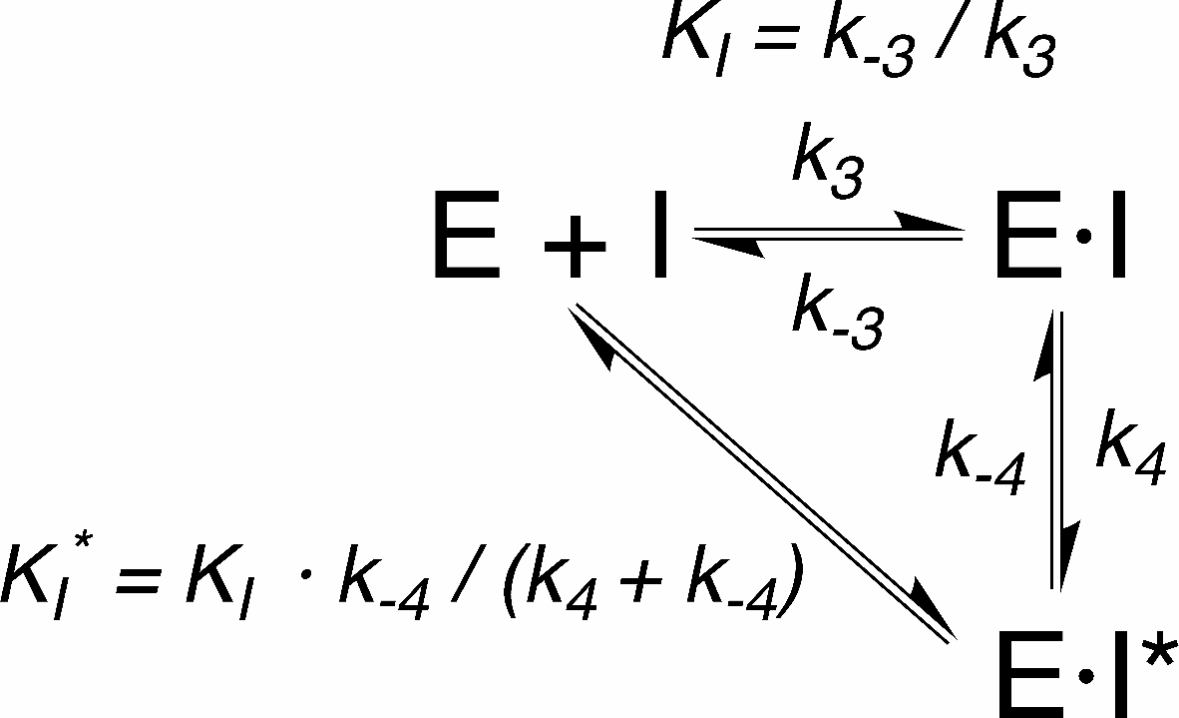
Enzymatic inhibition reaction.

This behavior is characteristic of a reversible inhibitor that binds to the enzyme with association and dissociation kinetic constants (*k*_*3*_ and *k*_*-3*_) governing the equilibrium between the enzyme, the inhibitor, and the enzyme-inhibitor (E•I) complex (Fig. 6). These constants are of the same order of magnitude as those (*k*_*1*_ and *k*_*-1*_) governing the equilibrium between the enzyme, the substrate, and the enzyme–substrate (E•S) complex (Fig. 3). The observed pattern also implies the presence of a subsequent slow process, governed by *k*_*4*_ and *k*_*-4*_ constants that are smaller than *k*_*1*_ and *k*_*-1*_. This latter step is commonly interpreted as a rearrangement of E•I to a tighter E•I* complex^46^. The dissociation constants of the E•I and E•I* complexes are denoted here as *K*_*I*_ and *K*_I_*

**Fig. 7:**
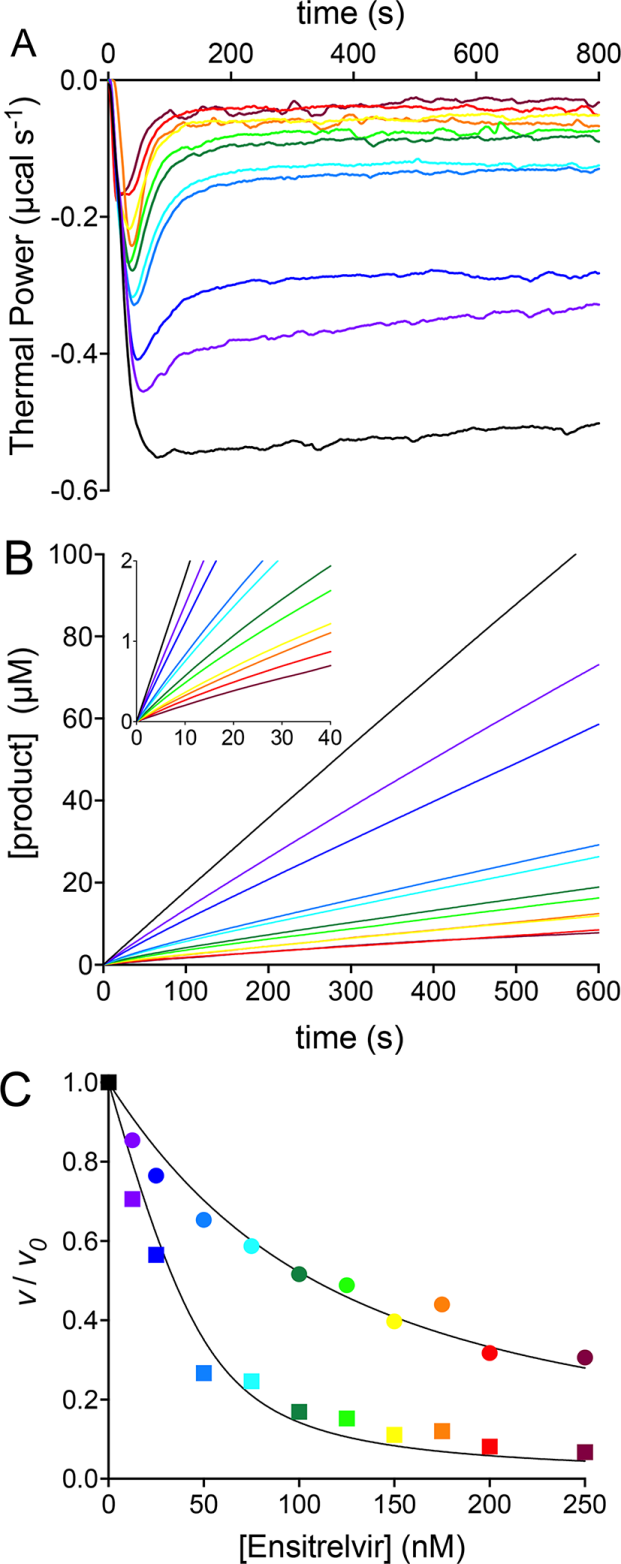
3CL^pro^ inhibition by Ensitrelvir at pH 7.5 and 298 K characterized by ITC. (**A**) Thermal power recorded over time following the injection of 50 nM 3CL^pro^ into the sample cell containing 0.60 mM substrate, in the absence of inhibitor (black) and supplemented with Ensitrelvir at concentrations of 12.5 nM (grape), 25 nM (blue), 50 nM (cyan), 75 nM (turquoise), 100 nM (dark green), 125 nM (light green), 150 nM (yellow), 175 nM (orange), 200 nM (red), 250 nM (maroon). (**B**) Progress curves derived from the raw data shown in (**A**); the inset shows the 0 – 40 s portion of each time course depicting the transition between the initial and the steady state phases. (**C**) *v*_*i*_ / *v*_*0*_ (circles) and *v*_*s*_ / *v*_*0*_ (squares) *vs.* Ensitrelvir concentration plots and corresponding fits to Eq. 8*.* In panels (**B**,**C**), the color-code corresponds to that in (**A**).

To analyze this behavior, we extracted progress curves depicting the increase of product concentration as a function of time at increasing Ensitrelvir concentrations (Fig. 7B). The use of ITC and the *inverse single injection method* to obtain progress curves has been previously reported^47–49^. The progress curve recorded in the absence of inhibitor lies on a straight line with a slope corresponding to the 3CL^pro^ reaction rate (*v*_*0*_). All progress curves in the presence of Ensitrelvir show an initial linear phase followed by a transition that eventually evolves toward a second linear phase with a smaller slope. The slope of the linear portions at early and late stages of each time course yielded the initial (*v*_*i*_) and the steady-state (*v*_*s*_) rate values, respectively. The steady-state rate is reached after the equilibrium involving E, I, E•I and E•I* has been fully established. Consistent with the inhibition mechanism reported in Fig. 6, both *v*_*i*_ and *v*_*s*_ progressively decreased in a concentration-dependent manner, corroborating the model. The sets of *v*_*i*_ and *v*_*s*_ values were used separately to quantitatively derive the apparent inhibition constants *K*_*I*_^*app*^ and K*_I_^*app*^, respectively for these two steps by using Eq. 8^46^ (Fig. 7C):8

Here, *[E]*_*T*_ and *[I]*_*T*_ are the total concentrations of enzyme and inhibitor, while *K*^*app*^ refers to either *K*_*i*_^*app*^ or to K*_i_^*app*^ depending on the use of *v*_*i*_ or *v*_*s*_ in place of v, respectively. *K*_*i*_^*app*^ and K*_i_^*app*^ for the two equilibria were derived as 83 ± 6 nM and 9.5 ± 1.7 nM, respectively. Assuming a competitive inhibition mechanism, as indicated by the X-ray structures of the 3CL^pro^-Ensitrelvir complex^42,50,51^, the inhibition constants (*K*_*I*_ and K*_I_) can be derived using Eq. 9^52^:9

The two distinct values thus obtained for *K*_*I*_ and K*_I_ were 9.9 ± 0.7 nM and 1.1 ± 0.2 nM.

## Discussion

This study represents the first successful application of isothermal titration calorimetry for characterizing the inhibition of the enzymatic activity of SARS-CoV-2 3CL^pro^ by Ensitrelvir, an anti-SARS-CoV-2 drug for COVID-19 selectively acting as a non-covalent inhibitor of this viral protease. ITC has proven to be an effective, rapid, and reliable method for obtaining kinetic and inhibition parameters. Using ITC, we can generate Michaelis–Menten profiles in under an hour. Our experimentation with different setups, including *direct multiple-injections* of substrate into enzyme solution and *inverse single-injection* of enzyme into substrate solution coupled with the use of progress curves, identified the latter as the optimal method for accurate kinetic and inhibition parameters determination.

The *inverse single-injection* approach using ITC offers several advantages over other previously employed assays like FRET or LC–MS for 3CL^pro^ kinetics and inhibition characterization. Unlike these methods, ITC continuously measures enzyme catalytic rates without requiring complex substrate or product concentration measurements in batch experiments. This approach allows for robust determination of Michaelis–Menten kinetics across a large number (hundreds) of substrate concentrations, avoiding the need for specific probes or post-reaction sample manipulations. The versatility of ITC is demonstrated by its ability to quickly provide the catalytic parameters *K*_*M*_ and *k*_*cat*_. The obtained value of *K*_*M*_ for peptide hydrolysis catalyzed by SARS-CoV-2 3CL^pro^ at 298 K (81 ± 2 µM) aligns more closely with FRET-derived values (28–230 µM^21,30^) rather than those obtained from LC–MS (0.9 mM^21^). On the other hand, the value of *k*_*cat*_ (3.9 ± 0.1 s^-1^) is more consistent with LC–MS values (2.2 s^-1^)^21^ rather than those from FRET (0.05–0.23 s^-1^^21,30,31^). Furthermore, the calorimetric assay provided the first experimentally determined activation energy (*E*_*a*_ = 13.1 ± 1.4 kcal mol^-1^) for 3CL^pro^ catalytic hydrolysis. This value is consistent with theoretical and experimental data for other cysteine proteases^53–55^, highlighting the robustness and novel insights enabled by ITC in enzyme kinetics studies. 

However, some potential issues and their resolutions must be discussed regarding the calorimetric assay used in this study. One possible concern is substrate or product inhibition. This was ruled out by analyzing the shape of the Michaelis–Menten curves and testing various substrate concentrations (0.15 – 0.35 mM), which showed no significant effect on *K*_*M*_ and *k*_*cat*_ (Fig. 1-SI). Another critical consideration is the presence of a residual concentration of 3CL^pro^ monomer in equilibrium with the dimer, as dimerization is essential for full enzymatic activity^8,9^. Reports of the dimer dissociation constant (*K*_*D*_) for SARS-CoV 3CL^pro^ range from 0.25 to 1 nM^20^, while values of *K*_*D*_ for the SARS-CoV-2 3CL^pro^ dimer vary more widely, ranging from 7 ± 1 μM (determined using small-angle X-ray scattering)^56^, to ~ 2.5 μM (from sedimentation velocities obtained by analytical ultracentrifugation)^57^, down to 0.14 ± 0.03 μM (from mass spectrometry data)^58^. In our approach, the enzyme concentration in the syringe is 15 µM, significantly higher than the above mentioned dissociation constants and much greater than concentrations used in FRET assays (0.1 – 2.0 µM^21,30^). It has been reported that the dissociation occurs over days at room temperature for SARS-CoV 3CL^pro^^59^ and is further disfavored in the presence of substrate^10^. Therefore, the *inverse single-injection* method used here likely maintains the enzyme in its active dimeric form as dilution from 15 µM in the syringe to 50 nM (or larger) in the sample cell occurs rapidly and in the presence of substrate. This was confirmed by the linear dependence of *V*_*max*_ as a function of a wide range of enzyme concentrations (0.050 – 1.2 µM, see Fig. 2-SI). This might explain the reason for the value of *k*_*cat*_ determined using ITC, (3.9 ± 0.1 s^-1^) being significantly higher than those determined by FRET and LC–MS methods, (0.05—2.2 s^-1^)^21,30,31^. Indeed, in the latter assays the reaction is initiated by the addition of the substrate into a solution containing the enzyme already equilibrated at the final concentration (in the absence of substrate), which might be so diluted that the enzyme could already be partially dissociated into the scarcely active monomeric form. The FRET-based assays also employed a wide range of enzyme-inhibitor incubation times (1.5 – 180 min) during which dissociation could occur at different degrees thus resulting in the high variability of the reported *k*_*cat*_ values. The E290A/R298A SARS-CoV-2 3CL^pro^ double mutant, a prevalently monomeric form of the enzyme^9^, was additionally tested using ITC and was indeed shown to be inactive (Fig. 3-SI), further indicating that the proposed experimental setup maintains 3CL^pro^ in a dimeric and fully active form.

The methodology adopted in this study to investigate the mode of action of Ensitrelvir as a non-covalent inhibitor of SARS-CoV-2 3CL^pro^ contrasts with the approach reported by Stille et al.^43^ to evaluate covalent inhibitors of the same enzyme using ITC. In our approach, the substrate concentration (0.60 mM) is deliberately set much higher than the estimated *K*_*M*_ value to maintain substrate saturation throughout the calorimetric measurement. At the same time, the enzyme concentration is kept low (0.05 µM) to reduce the hydrolysis rate, enabling the recording of ITC traces without substantial decrease in the reaction rate due to substrate depletion. As a result, any observed decrease in heat flow over time reflects only the interaction between the enzyme and the inhibitor. On the other hand, Stille et al*.*^43^ employed a lower substrate concentration (*ca*. 0.3 mM) and a significantly higher enzyme concentration (0.400 µM), leading to significant substrate depletion during the assay. Consequently, the reaction rates were affected by both substrate depletion and enzyme-inhibitor interactions, conditions that required complex data treatment involving convoluted algorithms to integrate differential equations and fit the results to obtain inhibition constants. 

The methodology described in this study offers significant advantages, particularly in distinguishing between fast and slow inhibitors, as well as between tight-binding, non-tight-binding, and covalent modes of action. By isolating the enzyme-inhibitor interaction from the complicating effects of substrate depletion, this method enables a detailed characterization of inhibition properties. Our ITC-based assay revealed that Ensitrelvir is a potent inhibitor of SARS-CoV-2 3CL^pro^ that acts as a tight- and slow-binding inhibitor, forming an initial E•I complex with a dissociation constant *K*_*I*_ = 9.9 ± 0.7 nM that evolves into a tighter E•I* complex with K*_I_ = 1.1 ± 0.2 nM within seconds. The key outcome of this study is therefore the identification of an initial step in the enzyme-inhibitor interaction, for which an estimate of its equilibrium constant can be inferred. This step is followed by a slower and tighter binding equilibrium, from which the enzyme-inhibitor affinity is also determined.

The previously reported affinity of Ensitrelvir for 3CL^pro^ in the absence of substrate resulted in the determination of a single dissociation constant *K*_*D*_ = 8.43 nM^45^ using ITC binding experiments, consistently with the *K*_*I*_ determined in this study for the E•I complex in the presence of substrate. The detection of an additional tighter equilibrium, with a dissociation constant of one order of magnitude smaller, suggests that the presence of substrate causes the enzyme to adopt a conformation that has higher affinity for the inhibitor in the E•I* complex.

ITC is the first method to directly estimate the 3CL^pro^ inhibition constants by Ensitrelvir, without the need to convert the IC_50_ values commonly derived from FRET^45,51,60^ and LC-MS^42^ assays via the Cheng-Prusoff equation (which implies the knowledge of the inhibition mode of the compound under study). The only reported value of *K*_*I*_ = 9 ± 0.7 nM for this inhibitor was indeed calculated from the IC_50_ value assuming a competitive mechanism^51^, consistently with the X-ray crystal structure reported for the enzyme-inhibitor complex^45,60^. In addition, while the enzyme-inhibitor incubation times used for FRET or LC–MS might determine the formation of a stable E•I complex prior to its exposure to the substrate, the described calorimetric assay provides a true competition experiment for the enzyme to either substrate or inhibitor.

## Conclusions

We propose the generalized use of the calorimetric assay developed in this study for investigating the kinetics of catalysis and inhibition of SARS-CoV-2 3CL^pro^. ITC can be used to validate novel hits from a 3CL^pro^ inhibitor high-throughput screen and to identify potential pan-assay interference compounds (PAINS) that can yield false positives in widely used high-throughput fluorescence-based platforms^61^. Moreover, ITC is invaluable not only for determining inhibition modes and relevant kinetic parameters but also for providing critical thermodynamic signatures, such as binding enthalpy and entropy, for protein–ligand interactions. These thermodynamic insights can significantly enhance drug design efforts toward improved 3CL^pro^ inhibitors. Specifically, focusing on optimizing enthalpic versus entropic contributions to binding for hit compounds has been shown to lead to better prioritization and optimization of ligands during hit selection and hit-to-lead processes. This approach can ultimately result in the development of more effective therapeutic agents^61,62^.

## Materials and methods

### Enzyme, substrate and inhibitor sources

Native 3CL^pro^ (M_r_ monomer = 33.8 kDa, pI = 5.95) was expressed using the plasmid vector pGTM_COV2_NSP5_004_SUMO (available from AddGene, ID: 190,062), and BL21(DE3) *Escherichia coli* cells, and purified following a previously described protocol^18^. The plasmid for the E290A/R298A 3CL^pro^ double mutant was obtained from GenScript (Rijswijk, Netherlands) starting from the above construct as a template. The E290A/R298A 3CL^pro^ double mutant was purified using the same procedure as for the native enzyme. 3CL^pro^ and its double mutant (purity > 98% as checked by SDS-PAGE) were stored as 150 µM and 900 µM stock aliquots, respectively (protein concentration is referred to the monomer throughout the manuscript) at -80 °C in 20 mM Tris–HCl buffer, 50 mM NaCl, 1 mM EDTA, at pH 7.5. The peptide substrate WKTSAVLQ/SGFRKMEW (M_r_ = 1.95 kDa, pI = 9.99) was designed with the 3CL^pro^ cleavage site Q/S, and was purchased from GenScript (Rijswijk, Netherlands). It includes two non-native tryptophan (W) residues at the N and C termini to ensure a measurable absorption at 280 nm. Solutions of substrate were freshly prepared before every experiment (see below for details). Protein and peptide quantification was carried out, prior to each experiment, by measuring the absorbance at 280 nm and considering a molar extinction coefficient (ε_280_) of 33,000 M^-1^ cm^-1^ and 11,000 M^-1^ cm^-1^ for 3CL^pro^ and the substrate respectively, estimated using ProtParam^63^. Ensitrelvir fumarate (molar mass = 647.95 g mol^-1^) was purchased from Cabru S.A.S. (Arcore, Italy), dissolved at 10 mM in pure DMSO, and stored as 10 µL aliquots at -80 °C.

### Calorimetric studies on the enzymatic hydrolysis by 3CL^pro^

The determination of the 3CL^pro^ kinetic parameters at fixed enzyme concentration was carried out using a high-sensitivity VP-ITC micro-calorimeter (MicroCal LLC, Northampton, MA, USA). For each experiment, the reference cell was filled with deionized water and the temperature of the reference and sample cells was set and stabilized at five temperatures in the range 298—310 K. Stirring speed was 300 rpm and the thermal power was monitored every 2 s using high instrumental feedback. Solutions of 3CL^pro^ for the assay were prepared by diluting a stock solution down to 15 µM in 600 µL of 20 mM Tris–HCl, 50 mM NaCl, 1 mM EDTA, 1% DMSO, at pH 7.5 (buffer A) and loaded into the injection syringe. The measured enzymatic activity was not affected over several days at 4 °C in the absence of DTT. The substrate was prepared by dissolving the purchased powder in 2 mL of the same buffer A, obtaining a final concentration of 0.35 mM, and loaded in the sample cell. The *inverse single-injection* experiment was carried out by injecting 15 µL of the 15 µM 3CL^pro^ solution (final enzyme concentration in the sample cell = 0.15 µM) from the syringe into the 0.35 mM substrate solution. The thermal power (TP, µcal s^-1^) was recorded over 2000 s, ensuring the instrument baseline shift caused by the heat flow generated from the enzymatic hydrolysis of the substrate to return to its original pre-injection level. The raw calorimetric data were processed using the *Method 1* available in the MicroCal Origin software to derive the total apparent molar enthalpy *ΔH*_*app*_ of 3CL^pro^-catalyzed substrate hydrolysis (according to Eq. 5). The reaction rates as a function of substrate concentration were obtained according to Eqs. 4 and 6, discarding the data obtained immediately after the injection of the substrate and before the calorimeter equilibrated with the ongoing reaction. The obtained reaction rates were fit using the canonical Michaelis–Menten equation (Eq. 1) to derive the kinetic parameters *K*_*M*_ and *k*_*cat*_. The determination of the 3CL^pro^ kinetic parameters at increasing monomer concentration (in the range 0.050 – 1.2 µM) was carried out at 298 K following the same procedure, using injection volumes in the 60 – 5 µL range. The same experimental setup was used for the E290A/R298A 3CL^pro^ double mutant by injecting 60 µL of a 900 µM protein (final concentration in the sample cell = 36 µM) into the sample cell containing 0.35 mM substrate. The experiments were performed as multiple technical replicates, each carried out with a minimum of three repetitions. The obtained values deviated by less than 5% across all measurements. The reported errors reflect the precision of the parameters obtained from a representative fit.

### Calorimetric studies on the enzymatic inhibition of 3CL^pro^ by Ensitrelvir

The determination of the inhibition parameters of Ensitrelvir on 3CL^pro^ was carried out using an *inverse single-injection* assay slightly modified with respect to that previously described. In this case, the 600-µL enzyme solution was prepared by diluting the stock 3CL^pro^ down to 5.0 µM using buffer A, while the 2-mL substrate solution was prepared at 0.60 mM in the same buffer. A first experiment was carried out by injecting 15 µL of the 3CL^pro^ solution (final enzyme concentration in the sample cell = 0.050 µM) into the substrate-containing sample cell. The use of smaller concentrations of enzyme and larger concentrations of substrate than those used for the Michaelis–Menten experiments ensured that, throughout the reaction time, the system fulfilled saturating and steady state conditions, thus ensuring a constant initial velocity. The thermal power was recorded over *ca.* 1000 s, a time that guaranteed the signal generated from the enzymatic hydrolysis of the substrate to be at a constant value, in turn signifying that the reaction rate is constant according to Eq. 4. A set of experiments was also carried out at increasing concentrations of Ensitrelvir in the 12.5 – 250 nM range, the latter being added by dilution of the 10 mM stock in buffer A to the substrate solution. This experimental setup therefore consisted in the injection of 15 µL of 3CL^pro^ in the sample cell (final enzyme concentration = 0.05 µM) that contained both the substrate and the inhibitor, thus providing a true competition experiment for the enzyme to either the substrate or the inhibitor. The recorded thermal power was integrated over time starting from the time at which it reached its minimum, the latter invariably occurring well after the nominal response time of the VP-ITC (10–20 s); the resulting total heat (expressed as µcal) was converted to the corresponding concentration of product formed during the reaction by considering *∆H*_*app*_ = -2.0 kcal mol^-1^. Taking into account the enzyme concentration, the sample cell volume, and the molar enthalpy for Ensitrelvir binding to 3CL^pro^ in the absence of substrate—previously determined to be -15.4 kcal/mol^64^—it can be estimated that the heat of inhibitor-enzyme binding is negligible compared to the overall heat measured in the assays, contributing approximately 1:2000. The obtained [P] formation was then plotted as a function of reaction time to yield progress curves. The initial (*v*_*i*_) and the steady-state (*v*_*s*_) rates in the presence of Ensitrelvir were derived by a linear fit of the first and last ten data points at early and late stages of each time course, respectively. The constant reaction rate in the absence of Ensitrelvir (*v*_*0*_) was derived as the slope of the corresponding linear progress curve. The obtained *v*_*i*_/*v*_*0*_ and *v*_*s*_/*v*_*0*_ values were used to derive the dissociation constants of the E•I and E•I* complexes according to Eqs. 8 and 9. Additional experiments, performed in the presence of fixed concentrations of Ensitrelvir (0.10 µM) and substrate (500 µM) and by injecting the enzyme solution (final concentration of 0.050 µM) at different injection rates (2.34 μL s^-1^, 1.17 μL s^-1^, and 0.59 μL s^-1^, corresponding to 5 s, 10 s, and 20 s of injection time), were carried out, with no significant variation of the derived parameters, thus ruling out a possible dependance of the injection rate (Fig. 4-SI). Finally, to consider possible effects of the instrumental response time on the accurate determination of the reaction rates, we estimated the inhibition constants by selectively removing the reaction rates determined at progressively increasing Ensitrelvir concentrations. The analysis of the results showed that the selective removal of the reaction rates does not significantly affect the result.

## Electronic supplementary material

Below is the link to the electronic supplementary material.

Supplementary Material 1

## Data Availability

The calorimetric raw data are available from the corresponding author upon request.
