# Author Correction: An isothermal calorimetry assay for determining steady state kinetic and Ensitrelvir inhibition parameters for SARS-CoV-2 3CL-protease

**DOI:** 10.1038/s41598-025-89345-x

**Published:** 2025-02-28

**Authors:** Luca Mazzei, Sofia Ranieri, Davide Silvestri, Rebecca Greene-Cramer, Christopher Cioffi, Gaetano T. Montelione, Stefano Ciurli

**Affiliations:** 1 Laboratory of Bioinorganic Chemistry, Department of Pharmacy and Biotechnology, University of Bologna, 40127 Bologna, Italy; 2 Center for Biotechnology and Interdisciplinary Sciences, Rensselaer Polytechnic Institute, Troy, NY 12180 USA; 3 Department of Chemistry and Chemical Biology, Rensselaer Polytechnic Institute, Troy, NY 12180 USA

Correction to: *Scientific Reports* 10.1038/s41598-024-81990-y, published online 31 December 2024

The original version of this Article contained an error in Equation 7, where the − symbol was missing,

$${k_{cat}}\propto {e^{\frac{{E_a}}{RT}}}$$

now reads:

$${k_{cat}}\propto {e^{-\frac{{E_a}}{RT}}}$$

In addition, Figure 6 also contained errors. The original Figure 6 and accompanying legend appear below.

**Fig. 6:**
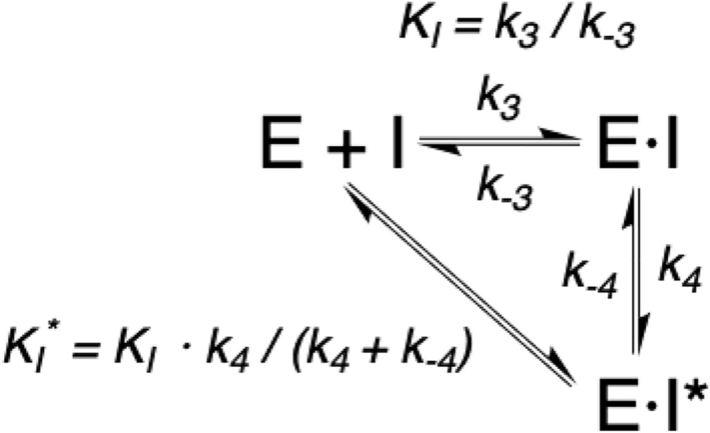
Enzymatic inhibition reaction.

The original Article has been corrected.

