# Supplementary material for: An isothermal calorimetry assay for determining steady state kinetic and Ensitrelvir inhibition parameters for SARS-CoV-2 3CL-protease
Source: Sci Rep. 2024 Dec 31;14:32175. doi: 10.1038/s41598-024-81990-y (PMC11688438; doi:10.1038/s41598-024-81990-y)
Supplement: Supplementary file 1 — Supplementary Material 1 [file 41598_2024_81990_MOESM1_ESM.pdf]

An isothermal calorimetry assay for determining steady state kinetic and Ensitrelvir inhibition parameters for SARS-CoV-2 3CL-protease

Luca Mazzei,[1,] Sofia Ranieri,[1] Davide Silvestri,[1] Rebecca Greene-Cramer,[2,3] Christopher Cioffi,[3]*

*Gaetano T. Montelione[2,3] and Stefano Ciurli[1,]**

[1]Laboratory of Bioinorganic Chemistry, Department of Pharmacy and Biotechnology, University of Bologna, I-40127 Italy

[2]Center for Biotechnology and Interdisciplinary Sciences, Rensselaer Polytechnic Institute, Troy, New York, 12180, USA

[3]Department of Chemistry and Chemical Biology, Rensselaer Polytechnic Institute, Troy, New York, 12180, USA

* Corresponding authors; email: luca.mazzei2@unibo.it; stefano.ciurli@unibo.it

Luca Mazzei	orcid: 0000-0003-1335-9365	e-mail: luca.mazzei2@unibo.it
Sofia Ranieri	orcid: 0009-0001-9046-4903	e-mail: sofia.ranieri2@studio.unibo.it
Davide Silvestri	orcid: 0009-0002-1852-5131	e-mail: davide.silvestri11@studio.unibo.it
Rebecca Greene-Cramer	orcid: 0000-0002-5967-1402	e-mail: greenr9@rpi.edu
Christopher Cioffi	orcid: 0000-0003-0642-7905	e-mail: cioffc4@rpi.edu
Gaetano T. Montelione	orcid: 0000-0002-9440-3059	e-mail: monteg3@rpi.edu
Stefano Ciurli	orcid: 0000-0001-9557-926X	e-mail: stefano.ciurli@unibo.it

SUPPLEMENTARY INFORMATION

Figure 1-SI. Superimposition of the Michaelis-Menten profiles determined for 150 nM 3CL^{pro} by using the *inverse single-injection* method with 0.35 mM (black curve) and 0.15 mM (red curve) substrate.

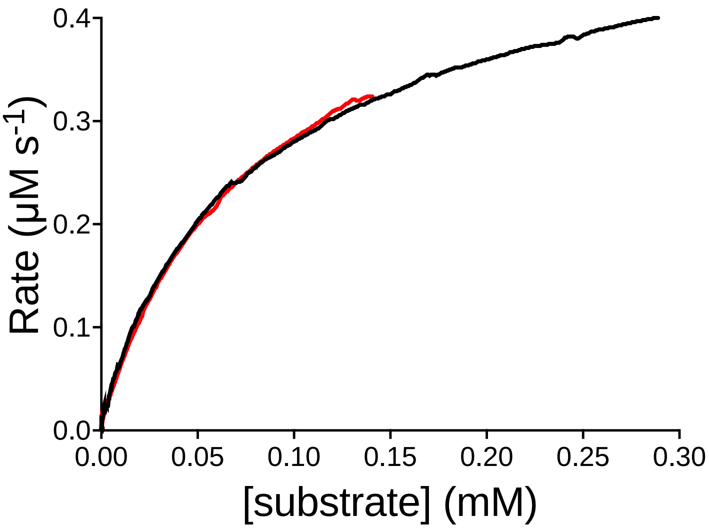

Figure 2-SI. Dependence of 3CL^{pro} V_{max} as a function of monomer concentration at pH 7.5.

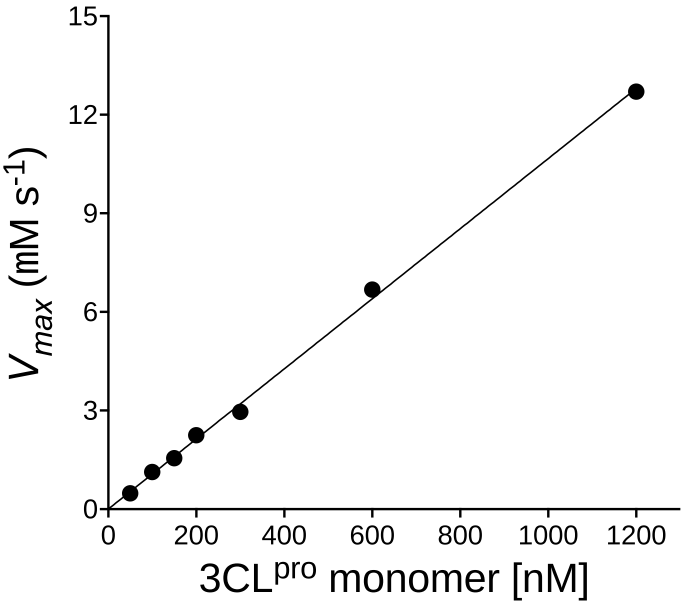

Figure 3-SI. Superimposition of the thermal power recorded over time corresponding to the enzyme activity of 150 nM (black) wild type $3CL^{pro}$ and its 36 µM E290A/R298A double mutant (red).

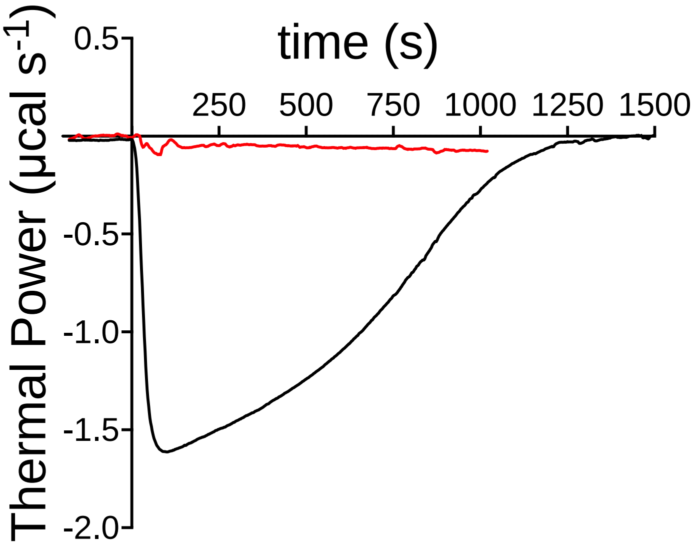

Figure 4-SI. Thermal power over time upon injection the enzyme solution (final concentration of 0.050 μM) to the sample cell containing a fixed concentrations of Ensitrelvir (0.10 μM) and substrate (0.60 mM) at the indicated injection rates (2.34 μL s^{-1}, 1.17 μL s^{-1}, and 0.59 μL s^{-1}, corresponding to 5 s, 10 s, and 20 s of injection time). A table reporting the derived values of v_i and v_s in all three cases is also shown.

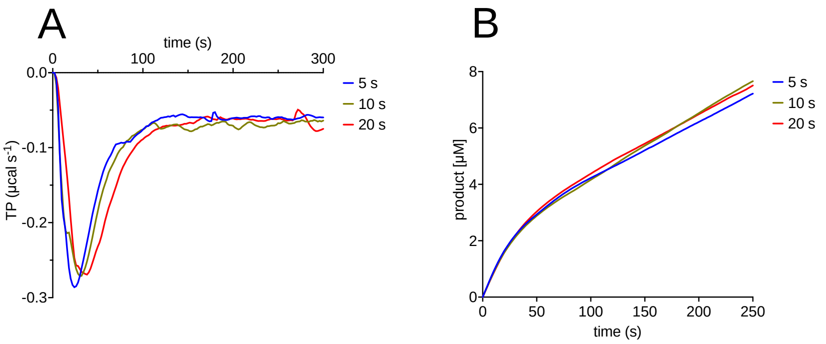

	5 s	10 s	20 s
***v*i**	0.2735	0.2615	0.2565
***v*s**	0.06181	0.06454	0.0651
